# Does ICSI for in vitro fertilization cause more aneuploid embryos?

**DOI:** 10.1186/s13039-020-00497-z

**Published:** 2020-07-01

**Authors:** Xiangli Niu, Jiamin Long, Fangqiang Gong, Weihua Wang

**Affiliations:** 1 Research Center for Reproductive Medicine, Reproductive Hospital of Guangxi Zhuang Autonomous Region, Nanning, Guangxi China; 2 Houston Fertility Laboratory, 2500 Fondren Rd., Suite 350, Houston, TX USA

**Keywords:** Oocytes, Regular IVF, Blastocysts, ICSI, Aneuploidy

## Abstract

**Background:**

High proportion of human embryos produced by in vitro fertilization (IVF) is aneuploidy. Many factors are related to the prevalence of embryonic aneuploidies, such as maternal age, sperm quality, and in vitro manipulation of oocytes. Oocytes are usually inseminated by intracytoplasmic sperm injection (ICSI) procedures for preimplantation genetic testing. There is still no available information whether insemination procedures, regular IVF or ICSI, affect embryonic aneuploidies.

**Methods:**

In this case report, a patient at her age of 47 years old received donated oocytes from a young donor for infertility treatment. Half of oocytes were inseminated by regular IVF and other half of oocytes were inseminated by ICSI. Fertilized oocytes were cultured to blastocyst stage and then biopsied for preimplantation genetic testing for aneuploidies (PGT-A). The proportions of aneuploidies were compared between two insemination procedures.

**Results:**

Forty-seven oocytes were retrieved, 23 were inseminated by regular IVF and 24 were removed from enclosed cumulus cells for ICSI. Out of 24 oocytes, 21 oocytes at metaphase II were inseminated by ICSI. After fertilization assessment, it was found that 12 oocytes from regular IVF fertilized normally. Nine blastocysts (75%) were biopsied and 1 (11.1%) was aneuploidy. By contrast, 19 out of 21 oocytes inseminated by ICSI fertilized normally, 14 blastocysts (73.7%) were obtained and 7 (50.0%) were aneuploidy. Transfer of a euploid blastocyst from regular IVF resulted in a healthy baby delivery.

**Conclusion:**

These results indicate that more embryos produced by ICSI are aneuploidy as compared with embryos produced by regular IVF. The results indicate that in vitro manipulation of oocytes for ICSI procedure may have adverse effect on human oocytes, and it may be one of the reasons causing aneuploid embryos in human IVF.

## Background

Preimplantation genetic testing for aneuploidies (PGT-A) has been widely applied in human in vitro fertilization (IVF), and has been one of embryo selection approaches apart from embryo morphology and time-lapse culture with morphokinetic embryo selection [1–7]. However, high proportion of human embryos produced by IVF is aneuploidy that cannot be revealed by morphological assessment and morphokinetic embryo selection, thus PGT-A is considered as a valuable procedure to screen embryos’ genetic status [1–5]. With PGT-A procedure, euploid embryos can be selected to transfer, which eventually can increase embryo implantation and reduce repeated implantation failures and birth defects [8–10].

Current PGT-A technology includes blastocyst biopsy and chromosomal screening by next generation sequencing (NGS) of whole chromosomes that can provide accurate chromosomal information including chromosomal number, chromosomal deletion and duplications [11–13]. Embryo biopsy at the blastocyst stage is a critical step to obtain samples for accurate chromosomal testing. For this purpose, oocyte insemination by intracytoplasmic sperm injection (ICSI) has been recommended as contamination by cumulus cells and/or sperm during regular IVF can be avoided [14].

Maternal age is one of the important factors affecting embryonic aneuploidy [15–27]. However, it has been found that aneuploidy rate in human IVF is quite high even when oocytes are collected from young patients or from young oocyte donors [15, 18–31]. The reasons for such high aneuploidy rates have not been addressed completely. Because most human aneuploid embryos are originated from oocytes [23, 32] and maternal age is an unquestionably factor to contribute to high aneuploidy rate in human embryos [15–19, 24]. Recently, a study reported that embryo aneuploidy rates from oocyte donor cycles are related to IVF laboratories [33]. Therefore, there must be other factors, except maternal ages, are related to embryo aneuploidy. One of the factors may be insemination procedures because oocyte second meiosis occurs during oocyte fertilization. Disruption of a normal meiosis can cause chromosomal errors during meiosis and eventually result in embryonic aneuploidy formation [23, 32, 34–36].

In the present case report, a patient received IVF treatment with donor oocytes with half of oocytes being inseminated by regular IVF and other half of oocytes being inseminated by ICSI. We found that different aneuploidy rates were present between two insemination procedures, thus we report this case and the information may be meaningful for physicians and clinical embryologists.

## Methods

### Donor stimulation for oocyte retrieval

The oocyte donor (34 years old) underwent controlled ovarian stimulation for 11 days with a combination of daily injection of 150 IU recombinant follicle-stimulating hormone (Gonal-F, EMD Serono, MA, USA) and 150 IU of a combination of follicle stimulating hormone and luteinizing hormone (Menopur, Ferring Pharmaceuticals, NJ, USA). On day 7, 0.25 mg gonadotropin releasing hormone antagonist (Cetrotide, EMD Serono) was given daily until triggering for oocyte maturation by 4 mg gonadotropin-releasing hormone agonist (Lupron) on Day 12 and 13. Oocytes were retrieved at 36 h after the first Lupron and then cultured in Global™Total medium at 37 °C in an atmosphere of 5.5% CO_2_, 6% O_2_, and balanced N_2_ under humidified conditions.

### Oocyte insemination by ICSI and regular IVF

For ICSI, cumulus cells were removed by using hyaluronidase (Fujifilm-Irvine Scientific, CA, USA) at 4 h after oocyte retrieval and metaphase II oocytes were injected 5 h after retrieval. For regular IVF, oocyte-cumulus complexes were inseminated directly in the organ culture dishes with 135,000 motile sperm/ml at 5 h after retrieval.

### Assessment of fertilization, embryo quality and blastocyst biopsy

Fertilization was assessed 18 h after insemination, and normal fertilization was characterized by two distinct pronuclei and two polar bodies. Embryo quality was evaluated by an inverted microscope on days 3, 5 and 6. Blastocysts at days 5 and 6 were biopsied using a modified inner zona biopsy method. Briefly, after holding the blastocyst to a proper position, a small hole in the zona was opened by the ZILOS-tk™ laser system (Hamilton Thorn Bioscience Inc., MA, USA) and a 20 μm polished biopsy pipette (Sunlight Medical, Jacksonville, FL, USA) was inserted inside zona through this hole. A few trophectoderm cells (5–10 cells) were aspirated into biopsy pipette inside the zona and then pull the pipette out of the zona. After assisted cutting (one or two pulses) with laser at the edge of the front opening of biopsy pipette, a fast mechanical friction between holding pipette and biopsy pipette was used to separate the cells from blastocyst. The biopsied cells were collected in PCR tubes and stored at − 20 °C freezer until processing for NGS. All blastocysts were cryopreserved for later frozen embryo transfer (FET).

### Chromosome analysis in the blastocysts

Biopsied samples were analyzed by a commercial genetic testing company (Invitea, San Francisco, CA, USA) using Illumina platform with a FAST-SeqS next generation of sequencing method and associated bioinformatics pipeline validated for accurate detection of whole chromosome number, segmental (≥10 Mb) aneuploidy, polyploidy and UPiD (chromosomes 1–16. 18, and X).

### Blastocyst vitrification, warming and transfer to recipient

The biopsied blastocysts were vitrified using a vitrification device and kit (Fujifilm-Irvine Scientific). Both equilibration solution and vitrification solution were warmed in original vials at 37 °C for at least 30 min before use. Briefly, collapsed blastocysts by a laser pulse were equilibrated in 100 μl drop of equilibration solution for 2 min, and then 45 s in 100 μl drop of vitrification solution (both steps were performed on a 37 °C warming stage) before loading to vitrification device. All blastocysts were vitrified individually and then stored in liquid nitrogen until warming for FET.

For warming, blastocyst was exposed to a thawing solution (Fujifilm-Irvine warming kit) at 37 °C for 1 min, transferred to a dilution solution for 3 min and finally to a washing solution for 10 min with a solution change after 5 min at room temperature. After completion of the warming process, blastocyst was cultured in Global™Total medium for 2 h before transfer.

For preparation of embryo transfer, the patient received 2 mg estradiol (Estrace, Warner Chilcott, NJ, USA) vaginally, 0.1 mg estradiol patch (Estradiol Transdermal System, Noven Pharmaceuticals, NJ, USA) every 3 days, and 400 mg progesterone (Cyclogest), twice a day, was administered on 15th day of estradiol treatment. The blastocyst was transferred on the sixth day of progesterone administered and progesterone was continued daily until the first serum β-hCG test 2 weeks after transfer. Ongoing pregnancy was supported by continued estradiol and progesterone until 11 weeks of pregnancy. Pregnancy was initially confirmed 14 days after embryo transfer by a serum β-hCG assay. Four weeks after embryo transfer, when a gestational sac and a heartbeat appeared, the patient was diagnosed as having a clinical pregnancy. The patient was then monitored by an obstetrician until childbirth.

## Case presentation

A 45 years old patient and her 44 years old male partner had 5 years infertility treatment with 10 previous failed IVF cycles. The male partner had normal semen analysis results (4 ml semen with 109 x10^6^sperm/ml and 41% motility). The patients decided to use donor oocytes for coming IVF treatment. Forty-seven oocytes were retrieved from an oocyte donor. Twenty three oocytes were inseminated by regular IVF and 21 oocytes at metaphase II (out of 24 oocytes) were inseminated by ICSI. As shown in Table 1, 16 oocytes inseminated by regular IVF and 19 oocytes inseminated by ICSI fertilized normally to form 2 pronuclei. All fertilized oocytes cleaved at Day 3 examination, and 9 and 14 blastocysts were obtained from regular IVF and ICSI, respectively. After PGT-A, it was found that 1 out of 9 blastocysts from regular IVF was aneuploid. However, 7 out of 14 blastocysts from ICSI were aneuploid.

**Table 1:** Fertilization and embryo development after regular IVF and ICSI

Insemination method	No. of oocytes	Oocytes at M-II^a^	Fertilization			No. of blastocysts	No. of aneuploidy
			0PN	3PN	2PN		
Regular IVF	23	NA	5	2	16 (69.6%)	9 (56.2%)	1 (11.1%)
ICSI	24	21	2	0	19 (90.5%)	14 (73.7%)	7 (50.0%)

^a^*M-II* metaphase II, *PN* Pronuclei

The details of chromosomal status and embryo quality of each blastocyst were shown in Table 2. It would appear that there is no relationship between embryo quality and chromosomal status of embryos. For example, 9 good blastocysts (both inner cell mass and trophectoderm) and 6 fare blastocysts (either inner cell mass or trophectoderm) were euploid while 6 good blastocysts and 2 fare blastocysts were aneuploid.

**Table 2:** Quality and chromosomal status of blastocysts from regular IVF and ICSI

Embryo #	Insemination method	Chromosomal Status	Blastocyst quality^a^	Embryo status
1	Regular IVF	46, XX	Good/Good	Transferred
2	Regular IVF	46, XX	Good/Good	Frozen
3	Regular IVF	46, XX	Good/Good	Frozen
4	Regular IVF	46, XX	Fare/Good	Frozen
5	Regular IVF	46, XY	Good/ Fare	Frozen
6	Regular IVF	46, XY	Good/Good	Frozen
7	Regular IVF	46, XY	Fare /Good	Frozen
8	Regular IVF	46, XY	Good/Good	Frozen
9	Regular IVF	47, XY, +16	Good/ Fare	Frozen
10	ICSI	46, XX	Good/Good	Frozen
11	ICSI	46, XX	Good/Good	Frozen
12	ICSI	46, XX	Fare /Good	Frozen
13	ICSI	46, XY	Fare /Good	Frozen
14	ICSI	46, XY	Good/Good	Frozen
15	ICSI	46, XY	Good/Good	Frozen
16	ICSI	46, XY	Fare /Good	Frozen
17	ICSI	45, XY,-16	Good/Good	Frozen
18	ICSI	48, XY, +3, +4	Good/Good	Frozen
19	ICSI	47, XY, +22	Good/Good	Frozen
20	ICSI	47, XX, +3	Good/Good	Frozen
21	ICSI	46, XY, del(7) (q32q34)	Good/Good	Frozen
22	ICSI	46, XY, del(3) (q21)	Fare /Good	Frozen
23	ICSI	46, XY, dup(7)(p11.2p22)	Good/Good	Frozen

^a^Inner cell mass/trophectoderm

After transfer of one euploid blastocyst (#1 embryo) resulting from regular IVF, the blastocyst implanted and a healthy girl (weight 3266 g) was delivered at gestation of 40 weeks and 5 days by cesarean section.

## Discussion

Application of PGT-A in human IVF has been increased significantly in recent few years and the benefits of PGT-A is to transfer euploid embryos that can increase embryo implantation and reduce birth defects. The reason for use of PGT-A is that high proportions of human embryos produced by IVF are aneuploidy that was found not only in patients with advanced maternal age [16, 20–22, 25, 37], but also in young patients [15, 28–30] or patients with donated oocytes for IVF [19, 31].

The origin of embryonic aneuploidies mainly is from oocytes by chromosomal errors during meiosis I and/or meiosis II [32, 34, 35]. Usually errors in meiosis I occur in in vivo during oocyte maturation, but errors during meiosis II occur during insemination in vitro [23, 32, 35]. Therefore, in vitro manipulation of oocyte and/or in vitro conditions may affect oocyte meiosis II. For PGT-A, oocytes are usually inseminated by ICSI, not by regular IVF, thus ICSI procedures (cumulus removal and ICSI procedure itself) increase more opportunities for oocytes to be exposed to sup-optimal conditions. The purpose for use of ICSI for PGT-A is to assure that cumulus cells around oocytes have been removed and only one sperm is used for insemination, so that there are limited cumulus cells attached to zona pellucida and no sperm around the oocyte, which can reduce the contamination of cumulus cells and/or sperm for more accurate genetic testing.

As a standard procedure for ICSI, cumulus cells need to be removed before ICSI and oocytes are inseminated after the oocytes are exposed to air under a controlled temperature conditions. Although ICSI has been used in human IVF for more than 30 years and live birth after ICSI and regular IVF did not show any differences [38, 39]. Very little information is available whether ICSI procedure can cause more oocytes to form aneuploid embryos because regular IVF is not used to inseminate oocytes if PGT-A is applied to the resulting embryos. In the present study, our case report, for the first time, indicates that ICSI procedures can cause more embryos to form aneuploidy as compared with regular IVF. The reason for this may be due to oocytes’ meiosis II process being exposed to a suboptimal in vitro conditions.

Temperature fluctuation is one of most critical factors affecting meiotic spindle organization which has been found in many mammals [40–45], and human oocytes are especially sensitive to temperature fluctuations [45]. It has been found that meiotic spindle in human oocyte depolymerized after the temperature dropped to 35 °C and spindles can recover after oocytes were returned to 37 °C, but limited recovery was also found in some oocytes [46–51].

Recently, it has also been reported that embryonic aneuploidy rates varied between physicians in same IVF laboratory and also varied among different clinics when oocytes were collected from young, healthy donors [33]. The reasons for these differences are unknown. Because many factors exist during IVF treatment, such as patient stimulation protocols, donor differences, different laboratory set up, laboratory environmental differences, and embryologist’s skill to perform ICSI. Our data from the present case may suggest that manipulation of oocytes in vitro including ICSI procedure itself may affect meiosis II, which eventually affect embryonic aneuploidy formation.

In the present case report, we used a new blastocyst biopsy method in which blastocysts were biopsied inside the zona pellucida. This method can be used to avoid any contamination of cumulus cells and sperm during regular IVF. Therefore, for insemination of oocytes in patients for PGT-A, it is not necessary to use ICSI procedures. We have used this new method to perform blastocyst biopsy during the past few years and found it is an easy to use biopsy procedure. The times for embryos to be exposed to air and the time for biopsy can be reduced by this method, and we also found that limited laser cutting is required during biopsy and excessive laser cutting application may damage embryos and biopsied cells.

Embryo morphology does not exactly represent chromosomal status in the embryos, which has been reported by use of different morphological assessments, including simple embryo morphological assessment or recently developed time-lapse morphokinetic embryo selection [51–53]. That is the reason that PGT-A is still the most valuable method to screen chromosomal status in human embryos [54–59], while embryo biopsy for PGT is one of the most challenged procedures in an IVF laboratory.

Although we manipulate human oocytes for ICSI under a well-controlled temperature and other conditions, it would appear that these conditions are not the optimal conditions for human oocytes. Further improvement is still necessary to avoid adverse effects on oocytes during in vitro manipulation of oocytes for ICSI.

Previous studies have examined cytogenetic results of spontaneous abortions following IVF and ICSI but the results were contradictory [60–62]. Lathi and Milki found that significantly higher aneuploidy rate in the abortuses of patients who conceived with ICSI than that with IVF [60], while other studies did not find the difference between regular IVF and ICSI [61, 62]. The different results from these studies may be resulted from sample collection. Many factors in these studies, such as the proportions of miscarriage out of total clinical pregnancy, were not known. Furthermore, only one sample was collected from each patient, so the chromosomal status in other embryos that have not been transferred in the same patients were not known either. While in the present case report, we analyzed all embryos produced by either regular IVF or ICSI from single oocyte retrieval cycle, thus the differences between patients, or between cycles were avoided, which makes the results to be more reliable.

## Conclusion

The present case report indicates that more human embryos produced by ICSI are aneuploidy as compared with the embryos produced by regular IVF. The higher aneuploidy rate may be related to in vitro manipulation of oocytes for ICSI including cumulus cell removal, ICSI procedure itself and/or temperature fluctuations during the processing. These processes may be suboptimal conditions for oocytes to undergo meiosis, therefore further improvement of in vitro conditions for ICSI procedure may be necessary. In addition, blastocyst biopsy can be performed inside zona pellucida to avoid contamination of cumulus cells and/or sperm for chromosomal analyses of biopsied cells, thus oocytes for PGT can be inseminated by regular IVF if semen analysis shows a normal sperm number, motility and morphology. Further comparison of aneuploidy rate in human embryos produced by regular IVF and ICSI remains necessary.

## Data Availability

The primary data for this study is available from corresponding author on reasonable request.

## Abbreviations

FET: Frozen embryo transfer
ICSI: Intracytoplasmic sperm injection
IVF: In vitro fertilization
PGT-A: Preimplantation genetic testing for aneuploidies
M-II: Metaphase II
PN: Pronuclei

## References

[1] Adler A, Lee HL, McCulloh DH, Ampeloquio E, Clarke-Williams M, Wertz BH (2014). Blastocyst culture selects for euploid embryos: comparison of blastomere and trophectoderm biopsies. Reprod BioMed Online.

[2] Cárdenas-Nieto D, Forero-Castro M, Moreno-Ortiz H, Lucena-Quevedo E, Cuzzi J, Esteban-Pérez C (2020). Analysis of a preimplantation genetic test for aneuploidies in embryos from Colombian couples: a report of cases. J Reprod Infertil.

[3] Subira J, Craig J, Turner K, Bevan A, Ohuma E, Veigh EM (2016). Grade of the inner cell mass, but not trophectoderm, predicts live birth in fresh blastocyst single transfers. Hum Fertil (Camb).

[4] Ravichandran K, Guzman L, Escudero T, Zheng XZ, Colls P, Jordan A (2016). Causes and estimated incidences of sexchromosome misdiagnosis in preimplantation genetic diagnosis of aneuploidy. Reprod BioMed Online.

[5] Minasi MG, Fiorentino F, Ruberti A, Biricik A, Cursio E, Cotroneo E (2017). Genetic diseases and aneuploidies can be detected with a single blastocyst biopsy: a successful clinical approach. Hum Reprod.

[6] Fishel S, Campbell A, Foad F, Davies L, Best L, Davis N (2019). Evolution of embryo selection for IVF from subjective morphology assessment to objective time-lapse algorithms improves chance of live birth. Reprod BioMed Online.

[7] Pribenszky C, Nilselid AM, Montag M (2017). Time-lapse culture with morphokinetic embryo selection improves pregnancy and live birth chances and reduces early pregnancy loss: a meta-analysis. Reprod BioMed Online.

[8] Pagidas K, Ying Y, Keefe D (2008). Predictive value of preimplantation genetic diagnosis for aneuploidy screening in repeated IVF-ET cycles among women with recurrent implantation failure. J Assist Reprod Genet.

[9] Simon AL, Kiehl M, Fischer E, Proctor JG, Bush MR, Givens C (2018). Pregnancy outcomes from more than 1,800 in vitro fertilization cycles with the use of 24-chromosome single-nucleotide polymorphism–based preimplantation genetic testing for aneuploidy. Fertil Steril.

[10] Kurahashi H, Kato T, Miyazaki J, Nishizawa H, Nishio E, Furukawa H (2016). Preimplantation genetic diagnosis/screening by comprehensive molecular testing. Reprod Med Biol.

[11] Cai Y, Ding M, Lin F, Diao Z, Zhang N, Sun H (2019). Evaluation of preimplantation genetic testing based on next-generation sequencing for balanced reciprocal translocation carriers. Reprod BioMed Online.

[12] Zheng H, Jin H, Liu L, Liu J, Wang WH (2015). Application of next-generation sequencing for 24-chromosome aneuploidy screening of human preimplantation embryos. Mol Cytogenet.

[13] Palmerola KL, Vitez SF, Amrane S, Fischer CP, Forman EJ (2019). Minimizing mosaicism: assessing the impact of fertilization method on rate of mosaicism after next-generation sequencing (NGS) preimplantation genetic testing for aneuploidy (PGT-A). J Assist Reprod Genet.

[14] Thornhill AR, de Die-Smulders CE, Geraedts JP, Harper JC, Harton GL, Lavery SA (2005). ESHRE PGD consortium ‘Best practice guidelines for clinical preimplantation genetic diagnosis (PGD) and preimplantation genetic screening (PGS)’. Hum Reprod.

[15] Fragouli E, Wells D, Doshi A, Gotts S, Harper JC, Delhanty JD (2006). Complete cytogenetic investigation of oocytes from a young cancer patient with the use of comparative genomic hybridisation reveals meiotic errors. Prenat Diagn.

[16] Pellestor F, Andreo B, Arnal F, Humeau C, Demaille J (2003). Maternal aging and chromosomal abnormalities: new data drawn from in vitro unfertilized human oocytes. Hum Genet.

[17] Scott RT, Upham KM, Forman EJ, Hong KH, Scott KL, Taylor D (2013). Blastocyst biopsy with comprehensive chromosome screening and fresh embryo transfer significantly increases in vitro fertilization implantation and delivery rates: a randomized controlled trial. Fertil Steril.

[18] Scott RT, Ferry K, Su J, Tao X, Scott K, Treff NR (2012). Comprehensive chromosome screening is highly predictive of the reproductive potential of human embryos: a prospective, blinded, nonselection study. Fertil Steril.

[19] Haddad G, Deng M, Wang CT, Witz C, Williams D, Griffith J (2015). Assessment of aneuploidy formation in human blastocysts resulting from donated eggs and the necessity of the embryos for aneuploidy screening. J Assist Reprod Genet.

[20] Jones KT (2008). Meiosis in oocytes: predisposition to aneuploidy and its increased incidence with age. Hum Reprod Update.

[21] Chiang T, Schultz RM, Lampson MA (2012). Meiotic origins of maternal age-related aneuploidy. Biol Reprod.

[22] Liu J, Wang W, Sun X, Liu L, Jin H, Li M (2012). DNA microarray reveals that high proportions of human blastocysts from women of advanced maternal age are aneuploidy and mosaic. Biol Reprod.

[23] Battaglia DE, Goodwin P, Klein NA, Soules MR (1996). Influence of maternal age on meiotic spindle assembly in oocytes from naturally cycling women. Hum Reprod.

[24] Franasiak JM, Forman EJ, Hong KH, Werner MD, Upham KM, Treff NR (2014). The nature of aneuploidy with increasing age of the female partner: a review of 15,169 consecutive trophectoderm biopsies evaluated with comprehensive chromosomal screening. Fertil Steril.

[25] Platteau P, Staessen C, Michiels A, Van Steirteghem A, Liebaers I, Devroey P (2005). Preimplantation genetic diagnosis for aneuploidy screening in women older than 37 years. Fertil Steril.

[26] Spandorfer SD, Chung PH, Kligman I, Liu HC, Davis OK, Rosenwaks Z (2000). An analysis of the effect of age on implantation rates. J Assist Reprod Genet.

[27] Forman EJ, Upham KM, Cheng M, Zhao T, Hong KH, Treff NR (2013). Comprehensive chromosome screening alters traditional morphology-based embryo selection: a prospective study of 100 consecutive cycles of planned fresh euploid blastocyst transfer. Fertil Steril.

[28] Baart EB, Martini E, van den Berg I, Macklon NS, Galjaard RJ, Fauser BC (2006). Preimplantation genetic screening reveals a high incidence of aneuploidy and mosaicism in embryos from young women undergoing IVF. Hum Reprod.

[29] Yang Z, Liu J, Collins J, Salem SA, Liu X, Lyle SS (2012). Selection of single blastocysts for fresh transfer via standard morphology assessment alone and with array CGH for good prognosis IVF patients: results from a randomized pilot study. Mol Cytogenet.

[30] Munne S, Sandalinas M, Magli C, Gianaroli L, Cohen J, Warburton D (2004). Increased rate of aneuploid embryos in young women with previous aneuploid conceptions. Prenat Diagn.

[31] Sills E, Li X, Frederick JL, Khoury CD, Potter DA (2014). Determining parental origin of embryo aneuploidy: analysis of genetic error observed in 305 embryos derived from anonymous donor oocyte IVF cycles. Mol Cytogenet.

[32] Coticchio G, Dal Canto M, Mignini Renzini M, Guglielmo MC, Brambillasca F, Turchi D (2015). Oocyte maturation: gamete-somatic cells interactions, meiotic resumption, cytoskeletal dynamics and cytoplasmic reorganization. Hum Reprod Update.

[33] Munné S, Alikani M, Ribustello L, Colls P, Martínez-Ortiz PA, McCulloh DH (2017). Referring physician group. Euploidy rates in donor egg cycles significantly differ between fertility centers. Hum Reprod.

[34] Hassold T, Hunt P (2001). To err (meiotically) is human: the genesis of human aneuploidy. Nat Rev Genet.

[35] Wang WH, Sun QY (2006). Meiotic spindle, spindle checkpoint and embryonic aneuploidy. Front Biosci.

[36] Bennabi I, Terret ME, Verlhac MH (2016). Meiotic spindle assembly and hromosome segregation in oocytes. J Cell Biol.

[37] Liang L, Wang CT, Sun X, Liu L, Li M, Witz C (2013). Identification of chromosomal errors in human preimplantation embryos with oligonucleotide DNA microarray. PLoS One.

[38] Hourvitz A, Pri-Paz S, Dor J, Seidman DS (2005). Neonatal and obstetric outcome of pregnancies conceived by ICSI or IVF. Reprod BioMed Online.

[39] Bonduelle M, Liebaers I, Deketelaere V, Derde MP, Camus M, Devroey P (2002). Neonatal data on a cohort of 2889 infants born after ICSI (1991-1999) and of 2995 infants born after IVF (1983-1999). Hum Reprod.

[40] Moor RM, Crosby IM (1985). Temperature-induced abnormalities in sheep oocytes during maturation. J Reprod Fertil.

[41] Pickering SJ, Johnson MH (1987). The influence of cooling on the organization of the meiotic spindle of the mouse oocyte. Hum Reprod.

[42] Aman RR, Parks JE (1994). Effects of cooling and rewarming on the meiotic spindle and chromosomes of in vitro-matured bovine oocytes. Biol Reprod.

[43] Ye J, Coleman J, Hunter MG, Craigon J, Campbell KH, Luck MR (2007). Physiological temperature variants and culture media modify meiotic progression and developmental potential of pig oocytes in vitro. Reproduction..

[44] Liu RH, Sun QY, Li YH, Jiao LH, Wang WH (2003). Effects of cooling on meiotic spindle structure and chromosome alignment within in vitro matured porcine oocytes. Mol Reprod Dev.

[45] Wu B, Tong J, Leibo SP (1999). Effects of cooling germinal vesicle-stage bovine oocytes on meiotic spindle formation following in vitro maturation. Mol Reprod Dev.

[46] Almeida PA, Bolton VN (1995). The effect of temperature fluctuations on the cytoskeletal organisation and chromosomal constitution of the human oocyte. Zygote..

[47] Pickering SJ, Braude PR, Johnson MH, Cant A, Currie J (1990). Transient cooling to room temperature can cause irreversible disruption of the meiotic spindle in the human oocyte. Fertil Steril.

[48] Sun XF, Wang WH, Keefe DL (2004). Overheating is detrimental tomeiotic spindles within in vitro matured human oocytes. Zygote.

[49] Wang WH, Meng L, Hackett RJ, Odenbourg R, Keefe DL (2001). Limited recovery of meiotic spindles in living human oocytesafter cooling-rewarming observed using polarized light microscopy. Hum Reprod.

[50] Wang WH, Meng L, Hackett RJ, Oldenbourg R, Keefe DL (2002). Rigorous thermal control during intracytoplasmic sperm injection stabilizes the meiotic spindle and improves fertilization and pregnancy rates. Fertil Steril.

[51] Campbell A, Fishel S, Bowman N, Duffy S, Sedler M, Hickman CF (2013). Modelling a risk classification of aneuploidy in human embryos using non-invasive morphokinetics. Reprod BioMed Online.

[52] Campbell A, Fishel S, Bowman N, Duffy S, Sedler M, Thornton S (2013). Retrospective analysis of outcomes after IVF using an aneuploidy risk model derived from time-lapse imaging without PGS. Reprod BioMed Online.

[53] Guerif F, Lemseffer M, Leger J, Bidault R, Cadoret V, Chavez C (2010). Does early morphology provide additional selection power to blastocyst selection for transfer?. Reprod BioMed Online.

[54] Ottolini C, Rienzi L, Capalbo A (2014). A cautionary note against embryo aneuploidy risk assessment using time-lapse imaging. Reprod BioMed Online.

[55] Forman EJ, Hong KH, Ferry KM, Tao X, Taylor D, Levy B (2013). In vitro fertilization with single euploid blastocyst transfer: a randomized controlled trial. Fertil Steril.

[56] Munne S, Chen S, Colls P, Garrisi J, Zheng X, Cekleniak N (2007). Maternal age, morphology, development and chromosome abnormalities in over 6000 cleavage-stage embryos. Reprod BioMed Online.

[57] Minasi MG, Colasante A, Riccio T, Ruberti A, Casciani V, Scarselli F (2016). Correlation between aneuploidy, standard morphology evaluation and morphokinetic development in 1730 biopsied blastocysts: a consecutive case series study. Hum Reprod.

[58] Kaing A, Kroener LL, Tassin R, Li M, Liu L, Buyalos R (2018). Earlier day of blastocyst development is predictive of embryonic euploidy across all ages: essential data for physician decision-making and counseling patients. J Assist Reprod Genet.

[59] Lagalla C, Tarozzi N, Sciajno R, Wells D, Di Santo M, Nadalini M (2017). Embryos with morphokinetic abnormalities may develop into euploid blastocysts. Reprod BioMed Online.

[60] Lathi RB, Milki AA (2004). Rate of aneuploidy in miscarriages following in vitro fertilization and intracytoplasmic sperm injection. Fertil Steril.

[61] Ma S, Philipp T, Zhao Y, Stetten G, Robinson WP, Kalousek D (2006). Frequency of chromosomal abnormalities in spontaneous abortions derived from intracytoplasmic sperm injection compared with those from in vitro fertilization. Fertil Steril.

[62] Kushnir VA, Frattarelli JL (2009). Aneuploidy in abortuses following IVF and ICSI. J Assist Reprod Genet.

